# Sensitivity to Temporal Reward Structure in Amygdala Neurons

**DOI:** 10.1016/j.cub.2012.07.062

**Published:** 2012-10-09

**Authors:** Maria A. Bermudez, Carl Göbel, Wolfram Schultz

**Affiliations:** 1 Department of Physiology, Development and Neuroscience, University of Cambridge, Downing Street, Cambridge CB2 3DY, UK; 2 Department of Neurology, University Hospital Cologne, 50924 Cologne, Germany

## Abstract

The time of reward and the temporal structure of reward occurrence fundamentally influence behavioral reinforcement and decision processes [1–11]. However, despite knowledge about timing in sensory and motor systems [12–17], we know little about temporal mechanisms of neuronal reward processing. In this experiment, visual stimuli predicted different instantaneous probabilities of reward occurrence that resulted in specific temporal reward structures. Licking behavior demonstrated that the animals had developed expectations for the time of reward that reflected the instantaneous reward probabilities. Neurons in the amygdala, a major component of the brain's reward system [18–29], showed two types of reward signal, both of which were sensitive to the expected time of reward. First, the time courses of anticipatory activity preceding reward delivery followed the specific instantaneous reward probabilities and thus paralleled the temporal reward structures. Second, the magnitudes of responses following reward delivery covaried with the instantaneous reward probabilities, reflecting the influence of temporal reward structures at the moment of reward delivery. In being sensitive to temporal reward structure, the reward signals of amygdala neurons reflected the temporally specific expectations of reward. The data demonstrate an active involvement of amygdala neurons in timing processes that are crucial for reward function.

## Highlights

► Reward signals in amygdala neurons were exquisitely sensitive to reward timing ► Time courses of reward anticipatory activity paralleled the temporal reward structure ► Reward responses were modulated by instantaneous reward probability

## Results and Discussion

### Experimental Design

We studied the activity of single amygdala neurons in two rhesus monkeys using a Pavlovian reward prediction task superimposed on an ocular fixation task. We varied the instantaneous temporal reward probability, defined as the probability of reward occurring in the next time interval given that task progression is in the current interval. The instantaneous probability is the behaviorally relevant probability of obtaining a reward in the next time interval and thus determines the prediction of reward from moment to moment. The fact that the Pavlovian task did not allow the animal control over reward occurrence precluded confounds by operant behavioral responses.

**Figure 1:**
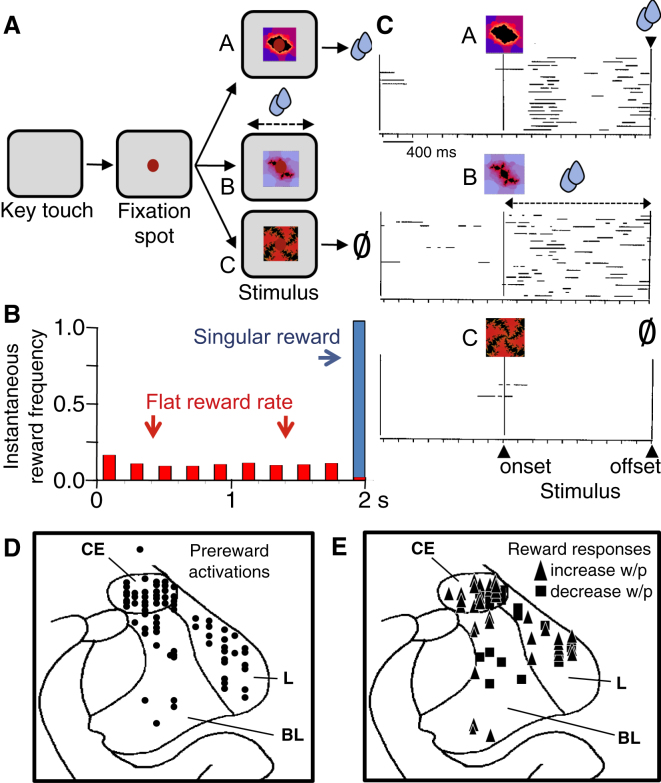
Task, Behavior and Recording Sites (A) Sequence of task events involving stimuli. Three pseudorandomly alternating stimuli predicted a singular reward with a probability of 1.0 at stimulus end (top), reward with a flat instantaneous reward probability of 0.025/50 ms interval during stimulus (middle), and no reward (bottom). (A fourth trial type, not shown, involved a flat instantaneous reward probability of 0.025/50 ms during the trial but without any stimulus.) (B) Measured instantaneous frequency of reward occurrence at stimulus end (blue, n = 1,045 trials) or with flat instantaneous probability (p = 0.025/50 ms) during stimulus (red, n = 646) (each vertical bar shows average from four intervals of 50 ms). Note that multiple rewards could occur during a single stimulus, thus producing flat moment-to-moment reward probability (analogous to “rate of occurrence of failure” for repairable systems rather than “hazard rate”). 0, stimulus onset. (C) Licking behavior in the three trial types shown in (A). Horizontal lines indicate photo beam interruptions by tongue at liquid spout. Each line shows one trial; trial sequence is from top to bottom. In middle graph, rewarded trials were excluded from analysis. (D and E) Histological reconstruction of recording sites in animal A, with approximate positions for animal B superimposed. (D) Location of neurons with prereward activations (n = 86 neurons). (E) Location of neurons with reward responses modulated by instantaneous reward probability. Triangles indicate higher responses with higher instantaneous reward probability (n = 36); squares show lower responses (n = 22). CE, central nucleus; L, lateral nucleus; BL, basolateral nucleus.

We varied instantaneous reward probability across four trial types. In one trial type, a singular reward occurred at the end of a specific 2.0 s visual stimulus (A; Figure 1A, top) with a probability of 1.0. Thus, instantaneous reward probability was zero (0) at all times during the stimulus and p = 1.0 at its end (Singular reward, Figure 1B, blue). In a second trial type, a different visual stimulus (B; Figure 1A, middle) predicted that reward would occur with an instantaneous probability of 0.025 in each interval of 50 ms during the entire stimulus duration of 2.0 s, but not in the absence of the stimulus. Thus, at any 50 ms interval during the stimulus, the probability that a reward would occur in the next 50 ms interval was 0.025. This probabilistic schedule allowed no reward or multiple rewards to occur during a single stimulus, which maintained the same flat instantaneous reward probability with flat reward rate during the entire stimulus duration (Figure 1B, red). In a third trial type, another visual stimulus (C) served as control without predicting any reward (Figure 1A, bottom). In a fourth trial type, no stimulus appeared, and reward occurred with a flat instantaneous probability of 0.025 in each 50 ms interval throughout 5.0 s of the 6.0 s trial cycle.

### Behavior

Both animals maintained key touch and central eye fixation in >95% of all trials with stimuli A–C throughout neuronal recordings. Error rates (erroneous key release) varied insignificantly between the rewarded trials but were significantly higher in the explicit no reward trial type (means of 8%–16% versus 27%–36% errors; p < 0.0001, one-way ANOVA; p < 0.01 for any rewarded trial type versus no reward trial type, Fisher's PLSD test; n = 86, 25, and 15 trial blocks during neuronal recordings of prereward and postreward activations). Anticipatory prestimulus licking was low (0 ms median lick duration across trials, in all three trial types) (Figure 1C). Careful adjustment of the licking spout with same distance between spout and mouth of the head-fixed animal resulted in similar licking in the two animals.

Stimulus A, which produced a singular reward at the fixed time of 2.0 s after stimulus onset, elicited licking in two periods, as observed before [30]. One peak occurred around 500 ms after stimulus onset as behavioral response to the stimulus, and a second peak occurred around 300 ms in anticipation of the time of the reward (Figure 1C, top; median lick duration/2 s, 473 ms). By contrast, stimulus B, which produced reward with flat instantaneous probability, elicited a lower, more tonic rate of licking during stimulus presentation compatible with the more spread-out reward occurrence (Figure 1C, middle; rewarded trials excluded from analysis; median lick duration/2 s, 395 ms). Very little licking occurred in the explicit no reward trials with stimulus C (Figure 1C, bottom; median lick duration/2 s, 0 ms).

Lick durations differed significantly between prestimulus and stimulus periods (p < 0.0001; F(1,208) = 193; two-way ANOVA) and among the three trial types (p < 0.0001; F(2, 208) = 94). Post hoc analysis identified longer licking durations during both rewarded stimuli compared to the no reward stimulus (both p < 0.0001, Fisher's PLSD test). Although the two rewarded stimuli elicited different temporal licking patterns, the overall amount of licking during the entire periods of the two stimuli differed insignificantly (p = 0.34).

These data demonstrate that the animals distinguished between the different temporal structures of reward predictions. Their licking followed the temporal profiles of reward occurrence, which suggests temporally differentiated reward expectations. The singular reward with stimulus A induced an expectation at stimulus end, whereas the reward occurring with flat instantaneous probability during stimulus B was associated with longer and more tonic reward expectations.

### Activity Preceding Reward

We tested 312 amygdala neurons in the two animals (204 and 108 neurons, respectively) with the singular reward delivered at the end of the 2.0 s stimulus A. Of these, 86 (28%) showed significant prereward activations during stimulus A (p < 0.05, Wilcoxon test against prestimulus control period). We tested the 86 neurons with the three stimuli (A–C) in pseudorandom alternation but skipped the fourth trial type, which lacked the required prestimulus control period. The 86 neurons were located in the central nucleus (47 of 144 tested neurons), basolateral nucleus (17 of 90 tested neurons), and lateral nucleus (22 of 78 tested neurons) of amygdala (Figure 1D). The activated neurons were insignificantly distributed among these three amygdala nuclei (p > 0.05, chi-square test).

**Figure 2:**
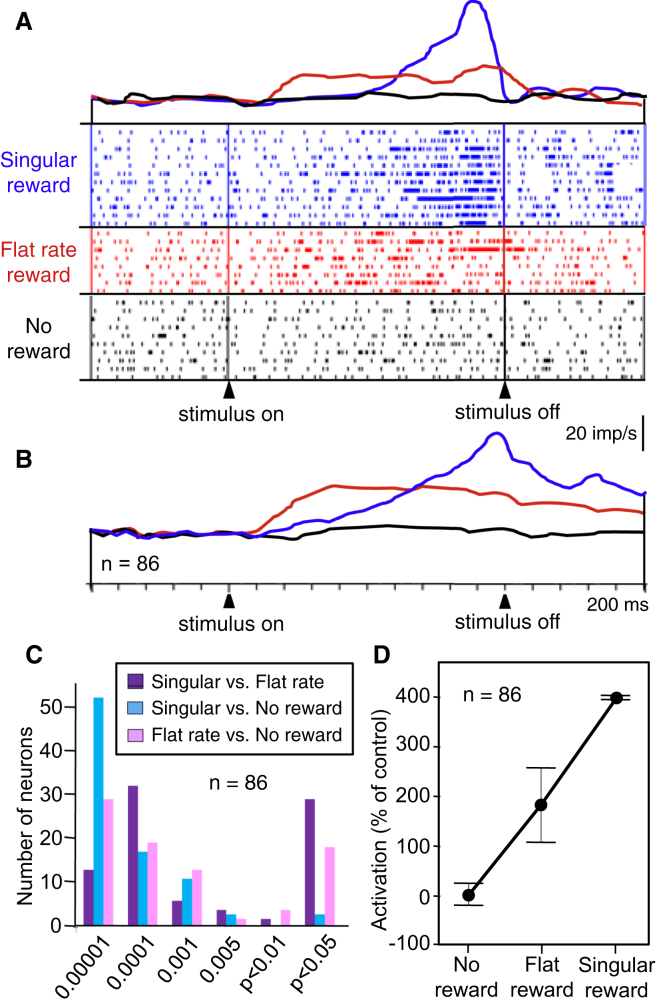
Modulation of Temporal Profiles of Neuronal Prereward Activity by Different Instantaneous Reward Probabilities (A) Single neuron. imp, impulse. (B) Population density functions of averaged activity elicited by the three stimuli predicting different instantaneous reward probabilities (blue, singular reward; red, flat reward rate during stimulus, rewarded trials excluded from analysis; black, no reward) (n = 86 neurons). Same bin width (10 ms) and impulses/s calibration bar apply to (A) and (B). (C) Distribution of neuronal p values from Fisher's PLSD post hoc two-sample comparisons following one-way ANOVA on prereward activations between singular, flat rate, and no reward trials. (D) Median activation strengths with different instantaneous reward probabilities (±95% confidence intervals).

The prereward activations in all 86 amygdala neurons were sensitive to reward timing. The activation of a typical neuron preceding the singular reward at the end of stimulus A is shown in Figure 2A (blue). It increased gradually after stimulus onset and dropped immediately when the reward was delivered after the fixed 2.0 s period at stimulus end. The average population activity shown in Figure 2B (blue) displayed a similar time course. The increase became significant against baseline at a mean of 1,200 ms (±54 ms SEM) after stimulus onset. By contrast, with flat instantaneous reward probability during stimulus B, activity increased earlier after stimulus onset compared to singular reward (mean latency, 350 ± 27 ms; p < 0.0001; n = 86; t test) and exceeded early activations with singular reward during 600–1,000 ms after stimulus onset [p < 0.0001, F(2,255) = 25.03, one-way ANOVA; p < 0.005, Fisher's PLSD on flat rate versus singular reward]. Activity remained tonically elevated during the remainder of the stimulus period (Figures 2A and 2B, red). (Note that rewarded trials were excluded from the main analysis and displays because of occasional confounding responses to the reward themselves; see below.) The activations failed to ramp up further during the stimulus and did not reach a clear peak (Figures 2A and 2B), despite the continuing increase in the sum of future reward. This temporal profile occurred also in the few trials in which the pseudorandom schedule produced several rewards. The differences in time courses of neuronal activity between singular reward and flat reward rate paralleled well the differences of the behavioral licking responses (compare Figures 2A and 2B with 1B, blue versus red).

Prereward activations were highest during the 400 ms window immediately preceding stimulus offset and significantly exceeded activations with flat instantaneous reward probability in the neuron of Figure 2A [p < 0.0001, F(2,27) = 12.64, one-way ANOVA; p = 0.0172, Fisher's PLSD on singular versus flat rate reward], in all 86 neurons analyzed individually, and in the population activity of the 86 neurons shown in Figure 2B [p < 0.0001, F(2,255) = 58.115, one-way ANOVA; p < 0.0001, Fisher's PLSD on singular versus flat rate reward and all other comparisons; Figure 2C]. During this 400 ms window, a Spearman correlation coefficient of rho = 0.615 and a comparison of activation strengths (Figure 2D) independently confirmed the neuronal sensitivity to instantaneous reward probability across explicit no reward, flat rate, and singular reward trials. Amygdala neurons showed bursts of impulses that increased toward the singular reward but were scattered throughout the occurrence of flat rate reward (Figure 2A, top and middle rasters). Defining bursts as greater than five impulses/100 ms, the overall burst rate varied insignificantly between singular and flat rate reward (means of 14.3 ± 4.8 SEM and 13.8 ± 3.9 bursts/ten trials, respectively; p > 0.5, t test). Bursts ended significantly later with singular compared to flat rate reward (472 ± 24.1 and 850 ± 47.3 ms before stimulus offset, respectively; p < 0.0001), which is compatible with the different temporal patterns of impulse rate with the two instantaneous reward rates. All 86 neurons failed to change activity during the no reward stimulus (p > 0.05, Wilcoxon test; Figures 2A and 2B, black).

Taken together, instantaneous reward probability had a remarkable influence on the temporal profiles of all prereward activations of the tested amygdala neurons. Whereas the neuronal activations preceding singular reward started late and reached high peaks, the activations with flat instantaneous reward probability started earlier and maintained a modest plateau until reward probability dropped with stimulus offset. The absence of ramping with flat reward rate during the stimulus suggested predictive coding of the instantaneous reward rate rather than coding of the increasing sum of future reward. Thus, the different temporal profiles reflected the different occurrences of predicted reward and constituted a typical characteristic of instantaneous reward expectation.

### Responses to Reward Delivery

Of the 312 amygdala neurons tested, 219 (70%) responded significantly to the reward delivered at the end of the 2.0 s stimulus (p < 0.05, Wilcoxon test against prereward control period, with exceptions stated below). We tested 169 of these neurons with all three rewarded trial types, and a subset of them with all four trial types (see below). Responses in 58 of the 169 neurons (34%) were sensitive to the instantaneous reward probability at the time of reward.

**Figure 3:**
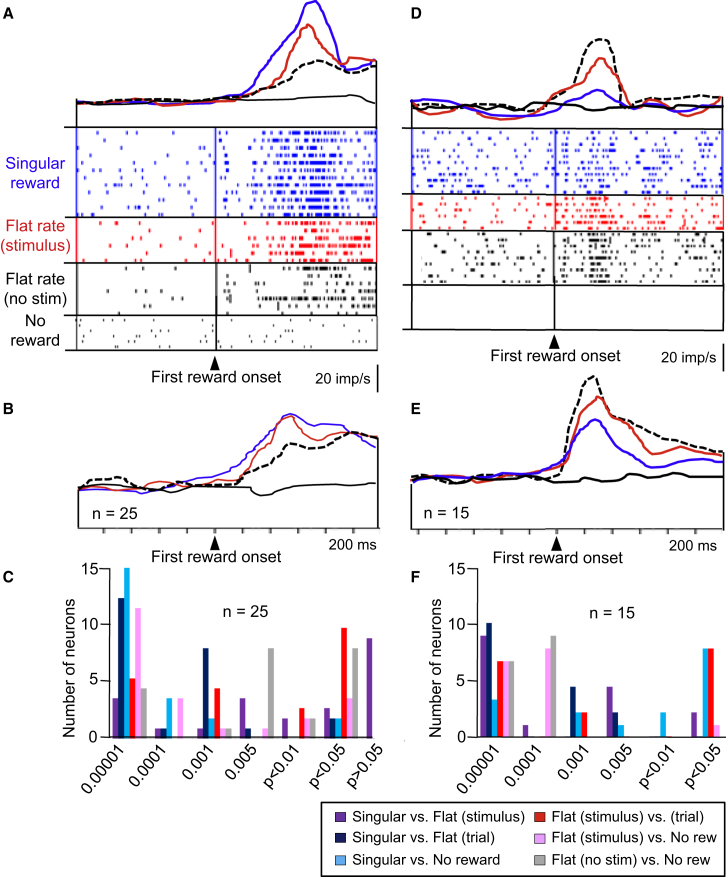
Increases of Neuronal Reward Responses to Onset of First Reward with Increasing Instantaneous Reward Probability (A) Single neuron. (B) Averaged population responses (n = 25 neurons). Same bin width (10 ms) and impulses/s calibration apply to (A) and (B). Color code in (A) and (B) same as for Figures 2A and 2B; dotted line indicates flat reward rate during entire trial without stimulus. (C) Distribution of p values from Fisher's PLSD post hoc test following one-way ANOVA. (D–F) The same as (A)–(C) but for decreases of neuronal reward responses with increasing instantaneous reward probability (n = 15 neurons).

Reward responses in 36 of the 169 neurons (21%) increased with increasing instantaneous reward probability. The 36 responding neurons were insignificantly distributed among the central, basolateral, and lateral amygdala nuclei (19, 8, and 9 of 95, 33, and 41 tested neurons, respectively; p > 0.05, chi-square test; Figure 1E, triangles). The responses were highest to the singular reward occurring after the fixed delay (Figure 3A, blue), lower with flat reward rate during the stimulus (red), lowest with flat reward rate during the trial (dotted), and absent in explicit no reward trials. The graded increases were seen in the neuron of Figure 3A [p < 0.0001, F(3,29) = 27.24, one-way ANOVA; p = 0.0075 singular versus flat rate during stimulus, p < 0.0301 flat rate during stimulus versus flat rate during trial, p < 0.0001 singular versus flat rate during trial; Fisher's PLSD test] and in all 36 neurons analyzed individually. The responses to flat rate reward during the stimulus varied only insignificantly between reward delivered during the first and the second half of stimulus duration (see Figure S1A available online; p > 0.1, t test). We tested 25 of these 36 neurons also in explicit no reward trials and found similar significant differences in the population activity of these neurons [Figure 3B; p < 0.0001, F(3,96) = 21.58, one-way ANOVA; p < 0.09 singular versus flat rate during stimulus, p < 0.001 flat rate during stimulus versus flat rate during trial, p < 0.0001 singular versus flat rate during trial, Fisher's PLSD test; Figure 3C]. Similar relationships were found in an additional 12 of 23 neurons that showed reward responses in addition to their prereward activations (the 23 neurons belonged to the group of 86 prereward neurons described above and were tested with a prestimulus control period).

Taken together, activity in these 36 amygdala neurons was positively modulated by instantaneous reward probability. The more likely the reward was to occur at any given moment, the higher was the neuronal response. Thus, the modulations varied positively with the temporal predictability of reward. The similarity of responses to flat rate reward during early and late stimulus periods suggested dependence on the predicted instantaneous reward rate rather than the predicted increasing sum of future reward. The observation that responses with flat reward rate without stimulus were lowest among all rewarded trial types would suggest that the stimulus had increased the predictability of the otherwise pseudorandomly timed reward. These activations might function to maintain established, temporally specific reward predictions after reinforcement learning. The positive relationship to instantaneous reward probability resembles the attentional modulation seen in monkey visual cortex V4, which parallels the hazard function of stimulus change during individual trials [12]. Our data demonstrate that such temporal modulations are not restricted to attentional processes but occur also with reward.

By contrast, reward responses in 22 of the 169 neurons (13%) decreased with increasing instantaneous reward probability. The 22 neurons were located in the central, basolateral, and lateral nuclei of amygdala (11, 5, and 6 of 95, 33, and 41 tested neurons, respectively; p > 0.05, chi-square test; Figure 1E, squares). The responses were absent in explicit no reward trials, lowest to singular reward (Figure 3D, blue), higher with flat reward rate during stimulus B (red), and highest with flat reward rate during the trial (dotted). This relationship was observed in the neuron of Figure 3D (p < 0.0001, F(3,30) = 17.78, one-way ANOVA; p = 0.0463 singular versus flat rate during stimulus, p = 0.0121 flat rate during stimulus versus flat rate during trial, p < 0.0001 singular versus flat rate during trial, Fisher's PLSD test) and in all 22 neurons analyzed individually. The responses to flat rate reward during the stimulus varied only insignificantly between rewards delivered during the first and the second half of stimulus duration (Figure S1B; p > 0.4, t test). We tested 15 of the 22 neurons also in explicit no reward trials and found similar significant differences in the average population activity of these neurons [Figure 3E; p < 0.0001, F(3,56) = 18.005, one-way ANOVA; p < 0.02 singular versus flat rate during stimulus, p < 0.02 flat rate during stimulus versus flat rate during trial, p < 0.0001 singular versus flat rate during trial, Fisher's PLSD test; Figure 3F]. The inverse relationship to instantaneous reward probability contrasted clearly with the positive relationship in the other neuronal group (Figure S2A). The responses in the two neuronal groups differed without overlap (Figure S2B).

Taken together, these 22 amygdala neurons showed an analogous but opposite instantaneous reward sensitivity to the 36 neurons described above. The less likely the reward was to occur at a given moment, the higher was the neuronal response, suggesting a relationship to temporal reward surprise. The similarity of responses to flat rate reward in the two stimulus periods suggested dependence on instantaneous reward rate rather than summed future reward. Such responses may reflect coding of positive temporal reward prediction errors, as observed previously [31, 32]. Further work may elucidate the nature and extent of time-sensitive prediction error responses of amygdala neurons.

### Conclusions

These data show that the temporal structure of reward occurrence influenced the activity of amygdala neurons. Although there was no explicit requirement to monitor reward timing, the animals' behavior reflected well the experimentally imposed instantaneous reward probabilities. The temporal statistics of reward occurrence modulated two forms of reward signal in different groups of amygdala neurons. Prereward activity paralleled the behaviorally expressed reward expectation. It ramped up to a singular reward at stimulus end but stayed at a lower, tonic level with reward dispersed over the whole stimulus period. These temporal profiles reveal an internal anticipatory process rather than simple build up of sensory responses over time and thus reflect a fundamental characteristic of reward expectation. Responses following the reward were enhanced when a reward was either more likely or more surprising to occur. Both types of activity reflected predictability of instantaneous reward rate rather than overall sum of future reward. These modulations suggest that amygdala neurons have access to an internal clock that processes the time of future reward occurrence. In being sensitive to reward timing, amygdala neurons process a fundamental characteristic of reward function and thus may play a more profound role in reward than hitherto known. Neuronal reward signals sensitive to the expected time of reward occurrence may be involved in a wide range of behavioral functions, including allocation of behavioral resources to specifically timed rewards, planning of sequential steps of goal-directed acts, choices between temporally distinct rewards, and assignment of credit to specifically timed reward during novel reward learning, value updating, and economic decision making, as conceptualized by animal learning and economic decision theories.

## Experimental Procedures

### Animals and Behavioral Task

Two adult male *Macaca mulatta* monkeys (4.4 and 6.7 kg) used before [28] served for the experiment. All procedures conformed to US National Institutes of Health Guidelines and were approved by the Ethical Review Committee of the University of Cambridge and the Home Office of the United Kingdom. Each trial started when the animal contacted a touch-sensitive key. Three trial types used visual stimuli A–C and eye fixation. A 1.3° ocular fixation spot appeared after key touch at the center of a computer monitor placed 450 mm in front of the animals. At 1,150 ms plus mean of 500 ms (truncated exponential distribution) after fixation spot onset, a single central 7° fractal visual stimulus appeared with the fixation spot superimposed (Figure 1A). An infrared optical system tracked eye position with 5 ms resolution (ISCAN). Stimulus and fixation spot extinguished together at 2.0 s after stimulus onset. Key release or fixation break during fixation spot presentation constituted an error and led to trial abortion and trial repetition. Intertrial periods lasted 4.0 s (from stimulus offset to onset of next stimulus). Thus, cycle time was 6.0 s (stimulus plus intertrial). The fourth trial type required key touch, did not use the fixation spot and specific stimuli, and had the same cycle time of 6.0 s.

The instantaneous reward probability states the probability with which a reward will be delivered in the next interval from the perspective of the current interval (see Supplemental Information for details). With stimulus A, a singular reward occurred with p = 1.0 at the fixed time of stimulus offset (Figures 1A and 1B), resulting in increases of instantaneous reward probability toward stimulus end. With stimulus B, instantaneous reward probability immediately increased after stimulus onset and subsequently was flat at p = 0.025/50 ms for the rest of the 2.0 s stimulus period. Stimulus C was not followed by any reward. These three trial types alternated pseudorandomly, sequences being limited to three consecutive same-trial types. In a fourth trial type, reward occurred with a flat instantaneous probability of 0.025/50 ms throughout 5.0 s of the 6.0 s trial cycle and without any stimulus (reward delivery stopped during 1.0 s for data storage and preparation of next trial, unannounced to the animal). To make this trial type detectable in the absence of any stimuli, it was run in separate blocks from the other three trial types. Because none, one, or several rewards could occur with the flat probability schedules, reward occurrence corresponded to the “rate of occurrence of failure” for repairable systems in reliability engineering [33]. An electromagnetic, computer-controlled liquid solenoid valve delivered identical magnitudes of individual reward (Ribena juice) in all rewarded trial types and emitted a noticeable, low-intensity click.

### Data Acquisition and Analysis

We recorded the activity of single neurons with single moveable microelectrodes and standard electrophysiological techniques during task performance while monitoring licking movements (see Supplemental Information for details). We used the paired Wilcoxon test on neuronal activity in each trial against control activity to assess onset and significance of prereward activations and postreward responses. We subsequently determined the influence of temporal reward structure on neuronal activity by comparing the Wilcoxon-identified activations between the different trial types with one-way ANOVA followed by Fisher's post hoc test and, independently, Spearman correlation (see Supplemental Information for details).

## Supplemental Information

Document S1. Figures S1 and S2, Supplemental Introduction, Supplemental Results, Supplemental Experimental Procedures, and Supplemental Discussion
