# Supplementary material for: Sensitivity to Temporal Reward Structure in Amygdala Neurons
Source: Curr Biol. 2012 Oct 9;22(19):1839–44. doi: 10.1016/j.cub.2012.07.062 (PMC3526777; doi:10.1016/j.cub.2012.07.062)
Supplement: Document S1. Figures S1 and S2, Supplemental Introduction, Supplemental Results, Supplemental Experimental Procedures, and Supplemental Discussion [file mmc1.pdf]

Current Biology, Volume *22*

Supplemental Information

Sensitivity to Temporal Reward Structure in Amygdala Neurons

Maria A. Bermudez, Carl Göbel, and Wolfram Schultz

Supplemental Inventory

1. Supplemental Figures and Tables

Figure S1

Figure S2

2. Supplemental Introduction

3. Supplemental Results

4. Supplemental Experimental Procedures

5. Supplemental Discussion

6. Supplemental References

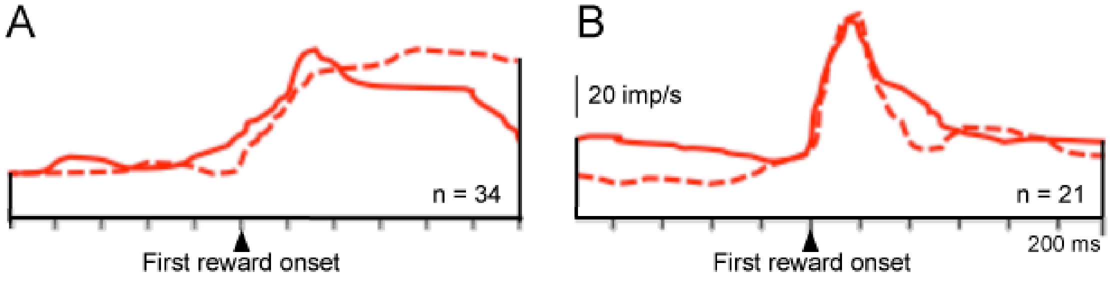

Figure S1. Lack of Dependence of Responses to Reward Delivery on Predicted Sum of Future Rewards

(**A**) Averaged population response in 34 neurons (1 trial per neuron), showing increased responses with increasing instantaneous reward probability. The responses failed to vary between first and last seconds of stimulus presentation (dotted and solid lines, respectively).

(**B**) Same as A, but for 22 neurons showing decreased responses with increasing instantaneous reward probability.

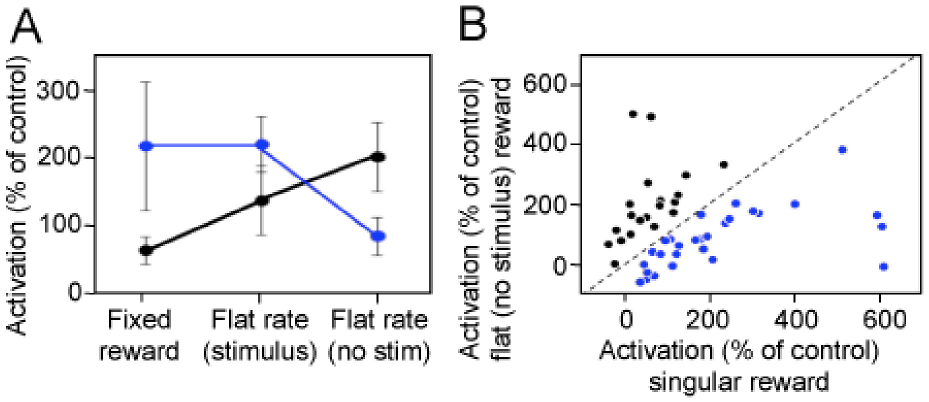

Figure S2. Quantitative Comparisons of Modulations of Neuronal Reward Responses by Instantaneous Reward Probability

(A) Median response strength modulated by instantaneous reward probability. Blue: responses with positive relationships; N=36 neurons. Black: negative relationships; N=22 neurons (±95 confidence intervals).

(B) Scatter plot demonstrating the effects of instantaneous reward probabilities. Blue: higher responses with singular reward than with flat reward rate during trial; N=34 neurons. Black: higher responses with flat reward rate during trial than with singular reward; N=20 neurons. Four neurons are not shown because of outlier (>750%) activations.

Supplemental Introduction

Time is of fundamental importance for behavioral reward processes. Humans and animals estimate time intervals with great accuracy [1, 2], which decays proportionally with delay but is timescale-invariant for constant delay:mean ratios (Weber-type, scalar expectancy theory; [3-5]). Reward intervals and rates are of crucial importance for conditioning and affect key learning variables such as contingency [5]. Theoretical reinforcement models are particularly efficient when taking time into account [6]. Conditioning requires optimal stimulus-reward intervals and becomes less efficient with shorter or longer intervals [7, 8]. Changes in reward timing induce learning and unblocking [9]. Subjective reward value is discounted with increasing delays to reward [10-12]. Thus, the crucial reward functions are highly sensitive to timing.

Despite the importance of timing and temporal structure for reward functions, we know little about underlying neuronal processes. Changes in dopamine neurotransmission following lesions or receptor binding drugs affect timing behavior [13-15], possibly reflecting an enabling function of dopamine. Neuronal responses in rat primary visual cortex vary with predicted reward delays [16]. Responses to reward predicting stimuli in cortical and subcortical reward neurons decrease with increasing delays [17-20], reflecting temporal discounting of reward value rather than specific temporal structure in reward predictions. Neurons in the striatum and several cortical areas show anticipatory activity that varies with instantaneous temporal probabilities of visual or somatosensory stimuli or movements [21-26]. However, it is unclear how these phenomena apply to reward processing. Anticipatory activity in thalamic, striatal and orbitofrontal neurons ramps up to the expected time of reward and is displaced by temporal shifts of reward [27-29]. Although suggesting sensitivity to reward timing, the displacements could result from delayed or slowed onsets of stimulus driven recruitment of neuronal activity. The only suggestion for sensitivity to temporal structure of reward predictions derives from the dopamine prediction error response elicited by temporally shifted rewards [30, 31], although this effect may be specific for error responses. Taken together, there is surprisingly little study of sensitivity to specific temporal reward structures.

The amygdala is a major component of the brain's reward system. Amygdala lesions disrupt behavioral reward processes and associated brain activations in humans [32-34] and impair several components of reward related behavior and reward learning in animals [35-39]. Amygdala neurons respond to reward predicting stimuli and reward delivery [40-45] and show slower activations preceding behavioral responses and reward [41, 46, 47].

Despite the importance of time in behavioral reward processes, and despite the demonstrated involvement of the amygdala in several reward processes, the temporal sensitivity of amygdala reward signals is unknown. The current study varied the instantaneous probability of reward occurrence in order to investigate the neuronal sensitivity to temporal reward structure. We tested the two principal and representative forms of amygdala reward signals, namely anticipatory activity preceding reward and responses to reward delivery. Our data suggest that both types of amygdala reward signal are sensitive to reward timing.

Supplemental Results

Responses to Reward Delivery

The responses to reward delivery with flat instantaneous reward rate during the stimulus varied only insignificantly between rewards delivered during the first second and the last second of the 2 s stimulus duration. This result refers to both activations increasing with

increasing instantaneous reward probability (Fig. S1A; P>0.1, t-test) and to activations decreasing with increasing instantaneous reward probability (Fig. S1B).

The differences in modulation by instantaneous reward probability in the two groups of amygdala neurons were confirmed by further quantitative comparisons. Both types of modulation showed high Spearman rank correlation coefficients (rho=0.725 and 0.851 for positive and negative relationships to instantaneous probability, respectively), but their slopes were opposite to each other (Fig. S2A blue vs. black). The separation of the two neuronal groups was also visible when plotting reward responses for flat reward rate against singular reward (Fig. S2B). Thus, instantaneous reward probability affected the responses to reward delivery in two distinct manners in the tested amygdala neurons.

Supplemental Experimental Procedures

Instantaneous Reward Probability

To achieve flat instantaneous reward probability during stimulus B, the computer advanced through the 2.0 s stimulus period in time steps of 50 ms and chose at every step an equally probable random number between 1 and 40; it marked that step when number 1 occurred. It delivered one unit of reward at every marked step. Thus no, one or several rewards could occur during a single stimulus. To avoid large variations, we recalculated reward occurrences for any 2.0 s stimulus period that contained more than 3 rewards. The delivered rewards summed to 10 within 10 trials. Thus, in any 50 ms interval during the 2 s stimulus period, the probability of receiving a reward in the next interval was p=0.025, which summed to p=1.0 in the 40 time steps during the stimulus. Through this procedure, the stimulus produced an extended, uniform, flat and constant instantaneous reward probability (Fig. 1B). In the fourth trial type without any stimulus, flat instantaneous reward probability was achieved in a similar manner, but the probabilty of p=0.025 applied to 5.0 s of the 6.0 s trial cycle.

Neuronal Recordings

After 5-7 months of behavioral training, a head holder and a recording chamber were fixed to the skull under general anesthesia and aseptic conditions. Recordings using tungsten microelectrodes and standard electrophysiological techniques and served to visualize impulses on oscilloscopes and transform them by threshold discrimination into binary electrical signals for 2 kHz sampling. We estimated the position of the amygdala from bone marks on frontal and lateral radiographs taken with an electrode guide cannula inserted at known coordinates relative to the stereotaxically implanted chamber [48]. Electrode positions were reconstructed in one animal from small electrolytic lesions (15-20 μA x 20-60 s) on 50 μm thick, cresyl violet-stained histological brain sections. As histological reconstruction was not available for the second animal for reasons of ongoing recordings, we reconstructed recording positions approximately from radiographic images. We collapsed recording sites from both monkeys spanning 3 mm in the anterior-posterior dimension onto the same coronal outline (Fig. 1D, E).

Data Acquisition and Analysis

Animals performed at least eight trials of each type for data acquisition (mean n=15 trials). We monitored licking movements by tongue interruptions of an infrared light beam at the liquid spout (STM Sensor Technology; 0.5 ms resolution). We assessed anticipatory licking as total durations of tongue interruptions during 2.0 s immediately preceding the stimuli and during the 2.0 s stimulus period and compared them between trial types (two-way Anova). To

avoid capturing reward reactions with flat instantaneous reward probability, we assessed licking only in trials in which the probabilistic schedule produced no reward.

We identified prereward activations in individual neurons by comparing activity increases between a fixed 400 ms time window immediately preceding reward onset and a standard 400 ms control period immediately preceding stimulus onset in the same trials with the nonparametric, one-tailed, signed-rank, matched-pairs Wilcoxon test ($P<0.05$). We expressed neuronal activations as percent of control period activity. We then compared the Wilcoxon-identified prereward activations with one-way Anova ($P<0.05$) across trial types using stimuli A-C. Fisher's PLSD posthoc test served to locate the activity differences ($P<0.05$). We used Spearman's rank correlation coefficient rho for additional, independent assessment of graded activity differences between trial types with differently timed rewards.

We used two measures to compare the speed of onset of the Wilcoxon-identified prereward activations between the different trial types. In the first measure we determined the latency of significant activations with a sliding time window procedure [49] which applied a Wilcoxon test ($P<0.01$) between a 100 ms time window and the constant 400 ms prestimulus control period. The window was moved in steps of 100 ms from stimulus onset through the stimulus period. We defined onset of activation at the center of the first 100 ms time window showing significant activation. In the second measure we compared activation strength in the interval of 600-1,000 ms after stimulus onset, during which amygdala neurons showed substantial activations with flat rate reward (Fig. 2A, B), using one-way Anova with Fisher's PLSD posthoc test. When analyzing data from trials with flat instantaneous reward probability, we considered only those trials in which the probabilistic reward schedule produced no rewards, to avoid contamination by potential reward responses.

We identified responses to reward delivery in individual neurons by comparing activity between a fixed 400 ms time window immediately following reward onset and the standard prestimulus 400 ms control window in the same trials with the Wilcoxon test ($P<0.05$). In trials using flat instantaneous reward probability without any stimulus, we aligned neuronal responses to the first reward after an initial period of 400 ms in each trial. We used the 400 ms prereward period as control period in these trials. We normalized the Wilcoxon-identified reward responses to control period activity and compared them with one-way Anova ($P<0.05$; followed by Fisher PLSD test) across all four trial types.

Control for Mouth Movements

We discarded two neurons whose activity showed temporal relationships to licking. These responses were closely related to tongue extension and retraction, resembling previously reported mouth movement-related activity in striatal neurons [50].

Supplemental Discussion

Task and Behaviour

The instantaneous reward probabilities imposed specific temporal reward structures and thus determined the temporal reward prediction during specific task epochs. Our instantaneous rate of occurrence of reward is analogous to the 'rate of occurrence of failure' (ROCOF) for repairable systems in reliability engineering [51] and reflects a discrete renewal process [52]. As with quickly repairable failures, our schedule allowed several rewards to occur during a stimulus or trial and thus defined reward prediction over the whole stimulus duration (stimulus B) or during the whole trial (fourth trial type). ROCOF differs from failure rate or hazard rate which refers to the conditional probability of event occurrence given that the

event has not yet occurred. Thus, failure or hazard rate would apply only to the first reward during the stimuli or trial and drop to zero afterwards, different from the current schedule.

Delivery of the singular reward at stimulus end is the usual way of reward delivery in most experiments. This temporal structure elicited licking during two periods. An early peak of licking followed the stimulus and constituted a simple conditioned response. A second phase of licking anticipated the time of reward and likely reflected the animals' reward expectation. By contrast, the flat instantaneous reward probability during the stimulus induced rather tonic anticipatory licking. Thus, the different licking patterns corresponded to the different temporal reward structures and likely reflected the animals' temporal reward expectations.

The current licking data corroborate earlier results suggesting behavioral sensitivity to temporal structure. During temporal discounting, anticipatory licking in monkeys peaks later with longer delays [19, 31]. During the temporal bisection procedure, monkeys distinguish well between two different, subsecond stimulus durations [23]. With movement triggering stimuli, ocular reaction times anticipate stimulus occurrence [26]. Thus, the current results are in line with previously reported behavioral sensitivity to temporal reward structure in monkeys.

Activity Preceding Reward

The experimentally imposed temporal structure affected the prereward activations of amygdala neurons in parallel with the behavioral licking responses. With singular reward, neuronal activity increased towards stimulus end, during a period in which anticipatory licking before the reward was highest. With flat instantaneous reward probability, the earlier, longer, more tonic and smaller increase of neuronal activity during most of the stimulus period paralleled the flat and low instantaneous reward probability and the prolonged anticipatory licking. The anticipatory activities did not just reflect anticipation of reward as such but, importantly, indicated when it would occur and at which rate. In the flat rate reward trials, the instantaneous rate of reward stayed constant during the stimulus whereas the sum of future rewards increased due to the probabilistic schedule. Thus, the flat neuronal activations during these trials seemed to reflect the prediction of the flat instantaneous reward rate rather than prediction of the sum of future rewards. These data suggest that these reward signals in the amygdala neurons have access to internal representations of temporal reward structure evoked by the specific reward predicting stimuli. In being sensitive to time, they process one of the most fundamental properties of reward expectation.

Our use of different temporal reward structures helps to resolve alternative interpretations. First, the different activities might simply reflect differences between the specific sensory stimuli predicting the two temporal structures. However, both the time courses and magnitudes covaried in a systematic and consistent fashion with the different imposed temporal structures and the resulting behavioral licking responses. None of the neurons showed a reverse relationship, namely sustained low activation with singular reward at stimulus end and ramping activity with flat instantaneous reward probability. These observations make a simple sensory relationship unlikely. Second, the gradual increase in neuronal activity toward the singular reward at stimulus end might result from a slow build up of neuronal activity induced by the stimulus. However, neuronal activity with flat reward rate paralleled the flat instantaneous reward probability and the corresponding licking behavior. These differences in time course argue against a neuronal build up induced by the stimulus. Third, reward rate was identical between the singular and flat reward schedules, thus making a simple relationship to value unlikely. Taken together, the observed temporal sensitivity of prereward activity reflected a typical characteristic of reward expectation.

Neuronal responses in monkey parietal cortex are sensitive to differently delayed discriminatory cues during temporal bisection [23] and show time dependent anticipatory activity that reflected the hazard rates of stimuli triggering eye movements [26]. Interestingly, time sensitive reward anticipatory activity occurred also in primary visual cortex [16]. Our data demonstrate temporal sensitivity of anticipatory activity in a typical reward structure. The combined evidence suggests that neurons may code the anticipated time for a range of behavioral events, such as sensory discrimination, movement and reward. However, it remains to be investigated whether these temporal influences derive from a central processor that operates independent of sensory discrimination, movement and reward. Alternatively, these functions may rely on their own timing devices.

Responses to Reward Delivery

The observed neuronal responses to reward delivery showed striking sensitivities to instantaneous reward probability in parallel with behavioral licking. The temporal modulations of these straightforward responses reflected the time varying strength of reward prediction at the moment of its delivery. The effects of reward predictability reflected the instantaneous reward rate, rather than sum of future rewards, but incorporated overall predictability, as shown by the differences in responses to flat rate rewards between presence and absence of stimulus. Together with the anticipatory coding of temporal reward expectation by prereward activations, the two major types of reward signals in the amygdala are highly sensitive to time.

Reward responses in one group of amygdala neurons were lowest to rewards delivered at flat instantaneous reward probability of p=0.025/50 ms over the entire trial, and highest to singular reward delivered with probability of p=1.0 at stimulus end. Responses with flat reward rate during the stimulus were intermediate between the two temporal profiles, possibly reflecting greater focus of reward expectation onto the stimulus period. Thus the modulations varied positively with the temporal predictability of reward. They might function to maintain established, temporally specific reward predictions after reinforcement learning. The positive relationship to instantaneous reward probability resembled the attentional modulation seen in monkey visual cortex V4 which paralleled the hazard function of stimulus change during individual trials [21]. Our data demonstrate that such temporal modulations are not restricted to attentional processes but occur also with rewards.

Reward responses in the second group of amygdala neurons showed the inverse relationship, increasing with lower instantaneous reward probability. They were lowest to the singular reward delivered with probability of p=1.0 at stimulus end, higher with rewards delivered with lower instantaneous reward probability during the stimulus, and highest with low probability rewards distributed over the entire trial. Such responses may reflect coding of positive temporal reward prediction errors, as observed previously [53, 54]. Although these studies did not address temporal relationships and our study did not test reward omission, the combined evidence suggests that time might influence the limited reward prediction error coding by amygdala neurons. The temporal influence may derive from dopamine projections [55] involved in interval timing [13, 15] and coding bidirectional reward prediction errors in a time sensitive manner [19, 30, 31]. The time specific reward responses are broadly analogous to the movement specific reward responses in dorsolateral and orbital prefrontal cortex [56], suggesting that reward signals in general may distinguish also between behavioural variables other than value and risk. Further work may elucidate the nature and extent of time sensitive prediction error responses of amygdala neurons.

Supplemental References

1. Church RM, Deluty MZ (1977) Bisection of temporal intervals. *J exp Psych: Anim Behav Proc* 3:216-228.
2. Roberts S (1981) Isolation of an internal clock. *J exp Psychol: Anim Behav Proc* 7:242-268.
3. Gibbon J (1977) Scalar expectancy theory and Weber's Law in animal timing. *Psychol Rev* 84: 279-325.
4. Rakitin BC, Gibbon J, Penney TB, Malapani C, Hinton SC, Meck WH (1998) Scalar expectancy theory and peak-interval timing in humans. *J Exp Psychol Anim Behav Proc* 24:15-33.
5. Gallistel CR, Gibbon J (2000) Time, rate and conditioning. *Psych Rev* 107:289-344.
6. Sutton RS, Barto AG (1998) *Reinforcement Learning* (MIT Press, Cambridge, MA).
7. Mackintosh NJ (1974) *The Psychology of Animal Learning* (Academic Press, London).
8. Wagner AR (1981) SOP: A model of automatic memory processing in animal behavior. In *Information Processing in Animals: Memory mechanisms*, eds Spear NE, Miller RR (Erlbaum, Hillsdale, NJ), pp 5-47.
9. Dickinson A, Hall G, Mackintosh NJ (1976) Surprise and the attenuation of blocking. *J exp Psychol: Anim Behav Proc* 2:313-322.
10. Ainslie G (1975) Specious rewards: a behavioral theory of impulsiveness and impulse control. *Psych Bull* 82:463-496.
11. Loewenstein G, Prelec D (1992) Anomalies in intertemporal choice: evidence and an interpretation. *Q J Econ* 107:573-597.
12. Ho M-Y, Mobini S, Chinang T-J, Bradshaw CM, Szabadi E 1999) Theory and method in the quantitative analysis of 'impulsive choice' behaviour: implications for psychopharmacology. *Psychopharmacology* 146:362-372.
13. Artieda J, Pastor MA, Lacuz F, Obeso JA (1992) Temporal discrimination is abnormal in Parkinson's disease. *Brain* 115:199-210.
14. Meck WH (1996) Neuropharmacology of timing and time perception. Cog Brain Res 3:227-242.
15. Buhusi CV, Meck WH (2005) What makes us tick? Functional and neural mechanisms of interval timing. *Nat Rev Neurosci* 6:755-765.
16. Shuler MG, Bear MF (2006) Reward timing in the primary visual cortex. *Science* 311:1606-1609.
17. Roesch MR, Olson CR (2005) Neuronal activity in orbitofrontal cortex reflects the value of time. *J Neurophysiol* 94:2457-2471.
18. Kim S, Hwang J, Lee D (2008) Prefrontal coding of temporally discounted values during intertemporal choice. *Neuron* 59:161–172.
19. Kobayashi S, Schultz W (2008) Influence of reward delays on responses of dopamine neurons. *J Neurosc*i 28:7837-7846.
20. Louie K, Glimcher PW (2010) Separating value from choice: delay discounting activity in the lateral intraparietal area. *J Neurosci* 30:5498-5507.
21. Ghose GM, Maunsell JHR (2002) Attentional modulation in visual cortex depends on task timing. *Nature* 419:616-620.
22. Brody CD, Hernandez A, Zainos A, Romo R (2003) Timing and neural encoding of somatosensory parametric working memory in macaque prefrontal cortex. Cereb Cortex 13:1196-1207.

23. Leon MI, Shadlen MN (2003) Representation of time by neurons in the posterior parietal cortex of the macaque. *Neuron* 38:317-327.
24. Matell MS, Meck WH, Nicolelis MAL (2003) Interval timing and the encoding of signal duration by ensembles of cortical and striatal neurons. *Behav Neurosci* 117:760-773.
25. Roux S, Coulmance M, Riehle A (2003) Context-related representation of time processes in monkey motor cortex. *Eur J Neurosci* 18:1011-1016.
26. Janssen P, Shadlen MN (2005) A representation of the hazard rate of elapsed time in macaque area LIP. *Nat Neurosci* 8: 234-241.
27. Schultz W, Apicella P, Scarnati E, Ljungberg T (1992) Neuronal activity in monkey ventral striatum related to the expectation of reward. *J Neurosci* 12:4595-4610.
28. Tremblay L, Schultz W (2000) Modifications of reward expectation-related neuronal activity during learning in primate orbitofrontal cortex. *J Neurophysiol* 83:1877-1885.
29. Komura Y, Tamura R, Uwano T, Nishijo H, Kaga K, Ono T (2001) Retrospective and prospective coding for predicted reward in the sensory thalamus. *Nature* 412:546-549.
30. Hollerman JR, Schultz W (1998) Dopamine neurons report an error in the temporal prediction of reward during learning. *Nat Neurosci* 1:304-309,
31. Fiorillo CD, Newsome WT, Schultz W (2008) The temporal precision of reward prediction in dopamine neurons. *Nat Neurosci* 11:966-973.
32. Bechara A, Damasio H, Damasio AR, Lee GP (1999) Different contributions of the human amygdala and ventromedial prefrontal cortex to decision-making. *J Neurosci* 19:5473–5481.
33. Johnsrude IS, Owen AM, White NM, Zhao WV, Bohbot V (2000). Impaired preference conditioning after anterior temporal lobe resection in humans. *J Neurosci* 20:2649–2656.
34. Hampton AN, Adolphs R, Tyszka JM, O’Doherty JP (2007) Contributions of the amygdala to reward expectancy and choice signals in human prefrontal cortex. *Neuron* 55:545-555.
35. Everitt BJ, Morris KA, O´Brien A, Robbins TW (1991) The basolateralamygdala-ventral striatal system and conditioned place preference: further evidence of limbic-striatal interactions underlying reward-related processes. *Neuroscience* 42:1-18.
36. Gaffan D, Murray EA, Fabre-Thorpe M (1993) Interaction of the amygdala with the frontal lobe in reward memory. *Eur J Neurosci* 5:968-975.
37. Malkova L, Gaffan D, Murray EA (1997) Excitotoxic lesions of the amygdala fail to produce impairment in visual learning for auditory secondary reinforcement but interfere with reinforcer devaluation effects in rhesus monkeys. *J Neurosci* 17:6011-6020.
38. Baxter MG, Parker A, Lindner CCC, Izquierdo AD, Murray EA (2000) Control of response selection by reinforcer value requires interaction of amygdala and orbital prefrontal cortex. *J Neurosci* 20:4311-4319.
39. Parkinson JA, Crofts HS, McGuigan M, Tomic DL, Everitt BJ, Roberts AC (2001) The role of primate amygdala in conditioned reinforcement. *J Neurosci* 21:7770-7780.
40. Sanghera MK, Rolls ET, Roper-Hall A (1979) Visual responses of neurons in the dorsolateral amygdala of the alert monkey. *Exp Neurol* 63:610-626.
41. Pratt WE, Mizumori SJY (1998) Characteristics of basolateral amygdala neuronal firing on a spatial memory task involving differential reward. *Behav Neurosci* 112:554-570.

42. Schoenbaum G, Chiba AA, Gallagher M (1999) Neural encoding in orbitofrontal cortex and basolateral amygdala during olfactory discrimination learning. *J Neurosci* 19:1876-1884.
43. Sugase-Miyamoto Y, Richmond BJ (2005) Neuronal signals in the monkey basolateral amygdala during reward schedules. *J Neurosci* 25:11071-11083.
44. Paton JJ, Belova MA, Morrison SE, Salzman CD (2006) The primate amygdala represents the positive and negative value of visual stimuli during learning. *Nature* 439:865-870.
45. Bermudez MA, Schultz W (2010) Responses of amygdala neurons to positive reward predicting stimuli depend on background reward (contingency) rather than stimulus-reward pairing (contiguity). *J Neurophysiol* 103:1158-1170.
46. Schoenbaum G, Chiba AA, Gallagher M (1998) Orbitofrontal cortex and basolateral amygdala encode expected outcomes during learning. *Nat Neurosci* 1:155-159.
47. Carelli RM, Williams JG, Hollander JA (2003) Basolateral amygdala neurons encode cocaine self-administration and cocaine-associated cues. *J Neurosci* 23:8204-8211.
48. Aggleton JP, Passingham RE (1981) Stereotaxic surgery under X-ray guidance in the rhesus monkey, with special reference to the amygdala. *Exp Brain Res* 44:271-276.
49. Schultz W, Romo R (1992) Role of primate basal ganglia and frontal cortex in the internal generation of movements: I. Preparatory activity in the anterior striatum. *Exp Brain Res* 91:363-384.
50. Apicella P, Ljungberg T, Scarnati E, Schultz W (1991) Responses to reward in monkey dorsal and ventral striatum. *Exp Brain Res* 85:491-500.
51. Luce RD (1986) *Response Times: Their Role in Inferring Elementary Mental Organization* (Oxford Univ Press, New York).
52. Doob JL (1948) Renewal theory from the point of view of the theory of probability. *Trans Am Math Soc* 63:422-438.
53. Belova MA, Paton JJ, Morrison SE, Salzman CD (2007) Expectation modulates neural responses to pleasant and aversive stimuli in primate amygdala. *Neuron* 55:970-984.
54. Roesch MR, Calu DJ, Esber GR, Schoenbaum G (2010) Neural correlates of variations in event processing during learning in basolateral amygdala. *J Neurosci* 30:2464-2471.
55. Sadikot AF, Parent A (1990) The monomaminergic innervation of the amygdala in the squirrel monkey: an immunohistological study. *Neuroscience* 36:431-447.
56. Tsujimoto S, Genovesio A, Wise SP (2009). Monkey orbitofrontal cortex encodes response choices near feedback time. *J Neurosci* 29:2569-2574.
